# Language Differences in the Brain Network for Reading in Naturalistic Story Reading and Lexical Decision

**DOI:** 10.1371/journal.pone.0124388

**Published:** 2015-05-27

**Authors:** Xiaojuan Wang, Jianfeng Yang, Jie Yang, W. Einar Mencl, Hua Shu, Jason David Zevin

**Affiliations:** 1 Key Lab for Behavior & Cognitive Neuroscience of Shaanxi Province, School of Psychology, Shaanxi Normal University, Xi’an, China; 2 State Key laboratory of Cognitive Neuroscience and Learning & IDG/McGovern Institute for Brain Research, Beijing Normal University, Beijing, China; 3 Institute of Psychology, Chinese Academy of Sciences, Beijing, China; 4 Department of Neurology, University of California Irvine, Irvine, California, United States of America; 5 Haskins Laboratories, Yale University, New Haven, CT, United States of America; 6 Departments of Psychology and Linguistics, University of Southern California, Los Angeles, CA, United States of America; University of Maryland, College Park, UNITED STATES

## Abstract

Differences in how writing systems represent language raise important questions about whether there could be a universal functional architecture for reading across languages. In order to study potential language differences in the neural networks that support reading skill, we collected fMRI data from readers of alphabetic (English) and morpho-syllabic (Chinese) writing systems during two reading tasks. In one, participants read short stories under conditions that approximate natural reading, and in the other, participants decided whether individual stimuli were real words or not. Prior work comparing these two writing systems has overwhelmingly used meta-linguistic tasks, generally supporting the conclusion that the reading system is organized differently for skilled readers of Chinese and English. We observed that language differences in the reading network were greatly dependent on task. In lexical decision, a pattern consistent with prior research was observed in which the Middle Frontal Gyrus (MFG) and right Fusiform Gyrus (rFFG) were more active for Chinese than for English, whereas the posterior temporal sulcus was more active for English than for Chinese. We found a very different pattern of language effects in a naturalistic reading paradigm, during which significant differences were only observed in visual regions not typically considered specific to the reading network, and the middle temporal gyrus, which is thought to be important for direct mapping of orthography to semantics. Indeed, in areas that are often discussed as supporting distinct cognitive or linguistic functions between the two languages, we observed interaction. Specifically, language differences were most pronounced in MFG and rFFG during the lexical decision task, whereas no language differences were observed in these areas during silent reading of text for comprehension.

## Introduction

Writing systems differ dramatically in how they represent language in written form: alphabets and syllabaries emphasize fidelity to the spoken forms of the language, whereas morphosyllabic systems combine probabilistic information about both sound and meaning [1–3]. Theoretical models also differ on whether distinct cognitive processes are required for reading these two kinds of writing system, as exemplified by models of English and Chinese.

One view holds that reading in English and Chinese involves the same set of mappings among orthographic (written) and phonological (spoken) forms of words and their semantics (meaning, [4]). Thus, the same basic processes are engaged by English and Chinese, but the "division of labor" [5] between them differs by degree. Consistent with this approach, we have shown that statistical learning models with the same functional architecture and learning rules simulate a range of effects in typical and disordered reading in both English and Chinese [6, 7].

Another view holds that English and Chinese writing systems differ qualitatively in the cognitive and neural processes they engage. English is characterized as involving the application of spelling-to-sound rules, so that the spoken form of a word can be "assembled" from its smaller, alphabetic parts (e.g., [8]). This process is associated with activity in the posterior superior temporal gyrus (pSTG, [9, 10]). Chinese, in contrast, is characterized as permitting only "addressed" phonology, or the retrieval from memory of whole syllables based on whole characters [3] a process further related to processing of non-linear spatial arrangements of orthographic forms, which is associated with activity in the left middle frontal gyrus (MFG, [11, 12]).

Thus far, language differences in the reading system have been observed entirely in tasks in which participants must make judgments about single words or pairs of words presented in isolation (e.g., [13–15]). Such tasks involve a host of ancillary meta-linguistic and decision processes that may determine how reading processes are flexibly deployed to meet task demands [16, 17] and that are unlikely to be engaged during reading under normal ecological conditions. Further, recent studies have demonstrated very large task by stimulus interactions throughout the reading system [18–20].

In the current study, we examined whether main effects of language on the organization of the reading system might be embedded in task by language interactions. We tested this by comparing activity in the reading network for English and Chinese under a naturalistic reading task and a commonly used artificial laboratory task (lexical decision). Data from the lexical decision task are consistent with prior comparisons between English and Chinese (e.g., the meta-analyses by [11, 21]). In contrast, data from the naturalistic reading task reveal a very different pattern of language. Language by task interactions are also observed in many regions, raising concerns that effects of language observed in studies of the reading system may need to be understood in the context of interactions between language properties and task demands.

## Materials and Methods

### Participants

Sixteen monolingual adults with normal vision participated in each experiment from each of two sites (one in the US, the other in China). Participants were matched across sites for age and education. They provided written informed consent and paid an hourly stipend. Each participant only performed one experimental task. The study was approved by ethical review boards at all sites (the Weill Cornell Medical College IRB and Yale's Humans Research Protection Program in US, IRB of BNU Imaging Center for Brain Research (BICBR) in China).

#### Experiment 1

Chinese speakers in Experiment 1 (8 males, 8 females) had a mean age of 21.8 (range: 18–25). English speakers in Experiment 1 (8 males, 8 females) had a mean age of 24.5 (range: 18–25). Data from Chinese speakers were collected at Beijing Normal University, and data from English speakers were collected at the Yale Magnetic Resonance Research Center.

#### Experiment 2

Chinese speakers in Experiment 2 (6 males, 10 females) had a mean age of 22.8 (range: 18–25). English speakers in Experiment 2 (6 males, 10 females) had a mean age of 23.6 (range: 19–39). Data from Chinese speakers were collected at Beijing Normal University, and data from English speakers were collected at Weill Cornell Medical College Citigroup Biomedical Imaging Center.

### Materials

#### Experiment 1

For the naturalistic reading task, six fairy tales (by Hans Christian Andersen) in Chinese translation were translated to English phrase by phrase, in order to match the timing of the experiment as closely as possible between languages. Each story had an average of 96 phrases with and average of 12 characters/words per phrase. A full story was presented in each run, and was split into 4 blocks, two for reading and two for listening task. Reading and listening blocks mixed in each run. Data from the periods during which the story was presented in spoken form are not included in the current analyses.

#### Experiment 2

For the lexical decision task, stimuli in Chinese comprised real characters, pseudo-characters and "artificial" character-like stimuli, with 30 stimuli in each condition designed to manipulate wordlikeness parametrically [22, 23]. The real characters were selected to be "phonograms", comprising a combination of a phonetic component that provides probabilistic information about pronunciation and a semantic component that provides probabilistic information about meaning. Three types of pseudo-characters were constructed, reflecting a parametric manipulation of wordlikeness: pseudo-characters containing both phonetic and semantic components, pseudo-characters containing only semantic components and pseudo-characters containing neither phonetic nor semantic components. Two types of artificial stimuli were also included, one in which the position of legal orthographic structures was reversed, violating orthotactic constraints of Chinese [24], and a stimulus type in which the position of strokes that make up a real character were randomly organized, destroying any larger-scale orthographic information. Ninety additional real character stimuli were included in order to balance the number of "word" responses in the lexical decision task. Filler frequency, alignment (left-right), number of radicals and strokes were matched to the target stimuli.

Stimuli for the English lexical decision task were also designed to vary parametrically in word-likeness. Thirty low-to-moderate frequency real words from four to six letters long were selected from the English Lexicon Project (http://elexicon.wustl.edu/default.asp), and a set of length- and bigram-frequency-matched pseudo-words was created using the same software. Two kinds of consonant string were created by combining either high-frequency bigrams (pairs of letters that occur frequently together such as "WR" and "GL") or low frequency bigrams, such as "KY" and "BK." Bigram frequencies were taken from Berndt, Reggia, and Mitchum[25]. Finally, a non-text condition was created by rearranging the strokes of individual letters to destroy any letter-level orthographic information. Thirty additional real-word stimuli—matched to the critical stimuli on length, frequency, and bigram frequency—were included as filler items. Note that although these are conceptually parallel manipulations, they necessarily differ in many details, making it impossible to examine effects of stimulus type across languages.

### Procedure

The same general procedures were used for both languages in each experiment. In both experiments, participants were familiarized with the task (for the lexical decision experiment, they were given a short practice session with different items), then lay comfortably in the scanner and viewed stimuli via rear projection.

#### Experiment 1

In the naturalistic reading experiments, participants read a story presented phrase by phrase at the center of the screen. Based on pilot work in which a comfortable reading rate was established, each phrase was presented for 2 seconds. Text was presented during long blocks of approximately one minute in length during which an average of 26 (range: 22–29) phrases were presented, yielding a mean block duration of 52s (range: 44-58s). The slight variability in block duration was introduced to avoid ending blocks mid-sentence. Each block was followed by 20s of rest, after which the story was continued in the auditory modality. Data from these listening blocks were not analyzed for the current report. After each story, a set of four multiple choice comprehension questions was presented.

#### Experiment 2

In the lexical decision experiments, individual stimuli were presented one at a time at the center of the screen, and participants were instructed to respond as quickly and accurately as possible, pushing a button with their right index finger for real words/characters, or their right middle finger to non-word/non-character stimuli. A fast random interval event- related design was used. On each trial, a 200ms fixation cross was presented, followed by a stimulus presented for 500ms, followed by a randomly jittered ITI (mean: 5.3s, range: 1-14s). The task was completed in two consecutive runs. Stimulus presentation was controlled, and response time and accuracy were recorded using E-Prime software.

Data from the lexical decision experiment in Chinese are taken from a study that also included a symbol detection task, and revealed task by stimulus interactions in this population [19], and data from the English lexical decision experiment are taken from a parallel study [26].

### MRI data acquisition

Functional and anatomical images were collected using 3T Siemens Magnetom TrioTim syngo MR systems, with 12-channel head coils, using identical data equipment and data collection parameters at all three sites. Functional images were collected using a gradient-recalled-echo echo-planar imaging sequence sensitive to the BOLD signal. Forty-one axial slices were collected with the following parameters: TR = 2500 ms, TE = 30 ms, flip angle = 90°, FOV = 20 cm, matrix = 64 x 64, 3mm thickness, yielding a voxel size of 3.125 x 3.125 x 3mm, interleaved slices with no gap. Following the acquisition of functional data, high resolution T1-weighted anatomical reference images were obtained using a 3D magnetization prepared rapid acquisition gradient echo (MPRAGE) sequence, TR = 2530 ms, TE = 3.45 ms, flip angle = 7°, FoV = 25.6 cm, matrix = 256 x 256 with 1 mm thick sagittal slices.

### MRI data analysis

Functional data were analyzed using AFNI ([27], program names appearing in parentheses below are part of the AFNI suite). Cortical surface models were created with FreeSurfer (available at http://surfer.nmr.mgh.harvard.edu/), and functional data projected into anatomical space using SUMA ([28 29], AFNI and SUMA are available at http://afni.nimh.nih.gov/afni).

Surface-based spatial normalization of anatomical and functional data was accomplished using Freesurfer [30] and SUMA [29]. Anatomical data were reconstructed (to3d), and a surface model for each participant was made with Freesurfer: cortical meshes were extracted from the structural volumes, and then inflated to a sphere and registered anatomically [30]. Using the surface atlas, an averaged subject was created by averaging surfaces, curvatures, and volumes from all participants both from Chinese and English. The averaged surface was converted into SUMA [29] as a standard mesh on the SUMA surfaces. The standard mesh was then converted to a volume and transformed to Talairach space (@auto_tlrc), using the N27 template [31] for visualization and reference purposes. Functional data were normalized by transforming volumes resulting from AFNI into surface representations using the standardized surfaces, and computing averages over surfaces. Reported Talairach coordinates are reported based on creating a 2x2x2mm AFNI volume from the average surface in each experiment (3dSurf2Vol).

### Preprocessing

The same preprocessing was conducted for data for both experiments. After reconstructing 3D AFNI datasets from 2D images (to3d), the anatomical and functional datasets for each participant were co-registered using positioning information from the scanner. The first 3 volumes were discarded, and functional datasets preprocessed to correct slice timing (3dTshift) and head movements (3dvolreg), reduce extreme values (3dDespike) and detrend linear and quadratic drifts (3dDetrend) from the time series of each run, with no smoothing or filtering.

### General linear models

Preprocessed data were analyzed in general linear model (GLM, 3dDeconvolve) for each experiment separately. The model for Experiment 1 included hypothetical hemodynamic response functions (HRFs) constructed by convolving the onsets and durations of reading blocks with a model HRF (waver) to test for task vs. rest effects, along with six regressors of no interest for head movement, and an additional regressor for the listening task. The model for Experiment 2 included a regressor constructed by convolving the onsets of trials in the lexical decision task with a gamma function HRF (3dDeconvolve), to compute task vs. rest effects, along with six regressors of no interest for head movement and an additional regressor for filler trials.

### Group analysis

Group analyses were conducted in the standard surface space [30]. In order to remove site effects from the data, FIRST-BIRN algorithms (from BXH/XCEDE tools (1.10.3) available at http://www.nitrc.org/projects/bxh_xcede_tools/) were used to estimate the signal- to-fluctuation-noise ratio (SFNR) for each participant’s entire data set, for inclusion as a covariate in all group-level analyses. This procedure has been shown to dramatically reduce effects introduced by variability between scanners at different sites [32]. Activation maps and regions reported as active in tables were obtained by first thresholding individual voxel at p <. 005 (uncorrected), and then applying a subsequent cluster-size threshold based on Monte Carlo simulations (AlphaSim), resulting in a corrected threshold of p <. 05.

### Region of Interest (ROI) selection for Language x Task analyses

In order to test for interactions between task and language, we selected four regions that are often discussed in the literature as distinguishing Chinese reading from English: left middle frontal gyrus (MFG), left posterior superior temporal gyrus and sulcus (pST) and a portion of ventral occipito-temporal cortex (vOT, or fusiform gyrus, FFG) associated with the processing of visual word form (in the left hemisphere) as well as its right hemisphere homologue. Regions of interest were taken from three meta-analyses: Tan et al.[11], Bolger et al.[21], and Wu et al.[33]. In the case of MFG, we observed that the same coordinates were identified as peak activations for Chinese readers in the Tan et al., and Wu et al. analyses. Tan et al. specifically observed that this region was not active for English readers, and discussed the implications of this finding at length (see also [34]). These coordinates were not identified as a peak in the Bolger et al. analyses, but activation in the same location, which was contiguous with activity in the inferior frontal gyrus, was identified in that study. Thus, we selected a single set of coordinates as most representative of the Chinese-specific MFG activation across studies.

In the case of left and right FFG, and left pST, multiple loci were identified across meta-analyses, raising the problem of how to create representative ROIs for these regions. In each case our first step was to collect all of the loci identified in the meta-analyses as corresponding to the gross anatomical location under consideration. We then created 6mm spheres around each locus, and combined voxels identified as overlapping across those loci to create ROIs. In Table 1, we present all of the loci used, data for both tasks and both languages from each, and indicate which overlapping regions were combined to create the ROIs.

**Table 1:** Regions identified from prior meta-analysis.

		Coordinates		
Region	Study	x	y	z
Left Fusiform Gyrus				
	Tan et al., 2005	-36	-54	-6
	Tan et al., 2005	-44	-54	-12
	Bolger et al., 2005	-37	-66	-18
	Bolger et al., 2005	-49	-58	-22
	Bolger et al., 2005	-37	-48	-22
	Bolger et al., 2005	-37	-76	-21
	Bolger et al., 2005	-49	-54	-15
	Bolger et al., 2005	-45	-58	-18
	Wu et al., 2012	-42	-70	-10
	Wu et al., 2012	-44	-60	-14
	Wu et al., 2012	-24	-90	-12
Right Fusiform Gyrus				
	Tan et al., 2005	34	-62	-18
	Bolger et al., 2005	33	-68	-21
	Wu et al., 2005	26	-92	-12
	Wu et al., 2005	44	-58	-12
Left Posterior Temporal				
	Tan et al., 2005	-56	-30	14
	Tan et al., 2005	-46	-34	0
	Bolger et al., 2005	-59	-51	6
	Bolger et al., 2005	-59	-45	15
	Bolger et al., 2005	-53	-31	9
	Bolger et al., 2005	-64	-22	1
	Bolger et al., 2005	-56	-28	4
	Wu et al., 2012	-58	-44	0

Notes: Coordinates(x, y and z) from previous meta-analysis are transformed into MNI atlas.

Each ROI was used as a mask to extract the mean coefficient (3dmaskave) for reading > rest contrast across languages and tasks. The task by language interaction was analyzed in each ROI via 2 x 2 ANCOVA, with SFNR for each participant as a covariate.

### Whole brain ANOVA analysis for Language x Task

To further identify brain regions for interaction between languages and task, each participant’s GLM dataset was mapped onto the averaged subjects’ surface. For each node of the surface, a language-by-task ANOVA analysis (3dANOVA2) was conducted. The resulting surface was mapped back (3dSurf2Vol) onto the volume dataset of averaged subject. The brain regions for language-by-task interaction were acquired by setting the threshold at voxel-wised p <. 005, and cluster-wise p <. 05.

## Results

### Experiment 1: Naturalistic reading

**Fig 1:**
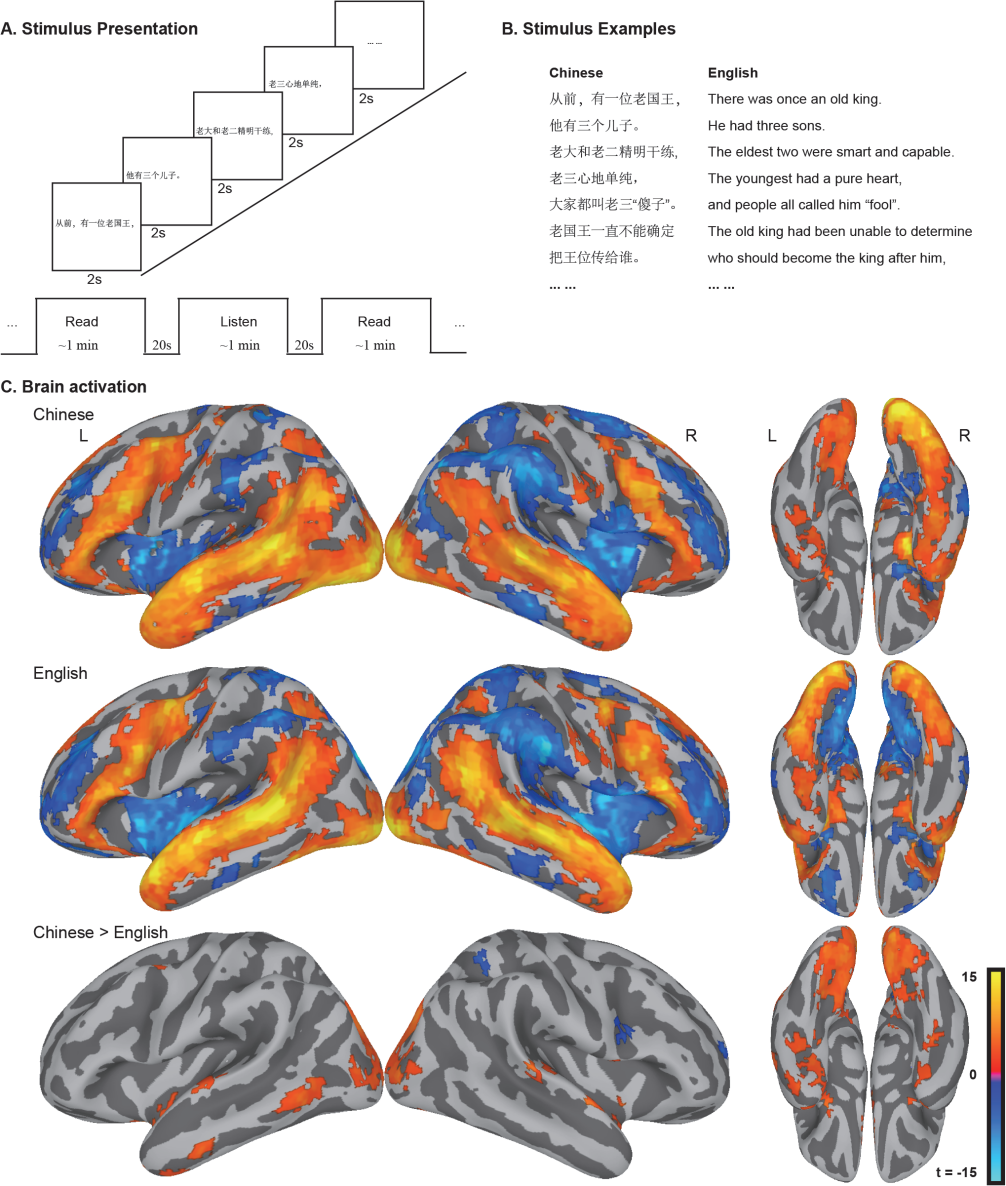
A. Stimuli were presented in intermixed blocks of reading and listening tasks. Each block lasted about one minute interspersed with 20s rest intervals. In reading block, 25 phrases (range 24–27) were presented continuously with each phrase lasting 2s; B. Examples of story materials. The Chinese story was translated into English phrase by phrase so that the narrative could be presented at the same rate for both groups. C. Very similar patterns of brain activity are engaged by naturalistic reading across languages (top, middle). The group contrast (bottom) revealed regions activated stronger for Chinese in visual cortex and regions for mapping spelling to meaning.

In order to compare activity between the two languages during naturalistic reading, matched groups of monolingual Chinese and English readers read and listened to a set of children’s stories in their respective languages while we collected fMRI data. Stories were presented phrase by phrase at a constant rate based on preliminary data from a self-paced reading task, as shown in Fig 1. Long (approximately one minute) blocks of reading were interspersed with equal intervals during which audio recordings of excerpts from the same story were presented (picking up where the printed text left off). Participants had no explicit task while reading, but were asked simple comprehension questions at the end of each story.

#### Task vs. rest contrast for each language

A general linear model (GLM) analysis of BOLD responses to reading compared to rest revealed highly similar maps across groups (Fig 1C, Table 2). For both scripts, robust activation was observed throughout visual regions in the occipital and temporal cortices bilaterally, in regions associated with semantic processing throughout the temporal lobe, and in temporal and frontal regions associated with phonological processing. Regions previously identified as playing a particular role in reading were identified in both groups, including the left FFG "visual word form area" [35, 36], a posterior middle temporal (pMTG) region associated with mapping from print to meaning [37 38], and a pST region associated with mapping from print to speech [9, 10]. Regions previously identified as specific to the Chinese reading network, such as the MFG and right FFG [11, 21, 33] were strongly activated for both languages.

**Table 2:** Regions identified in the Task > Rest contrast in Experiment 1.

					Coordinates		
Region	H	BA	Volume	t	x	y	z
Chinese Positive							
Frontal, Temporal, Fusiform	B		9640	32.40	14	-91	-13
Sup. Frontal Gyrus	B		1171	17.39	2	49	44
Medial Frontal Gyrus	R	11	265	12.34	2	49	-16
Precentral Gyrus	R	6	75	5.80	65	-6	28
	R	4	34	7.67	32	-32	60
	L	4	56	8.53	-11	-36	73
Postcentral Gyrus	L	3	82	9.38	-32	-35	56
Precuneus	R	19	44	7.89	32	-68	42
	R	19	41	5.56	32	-74	15
Thalamus			61	10.95	-4	-23	-2
Chinese Negative							
Cingulate Gyrus, Inf. Parietal	B		4437	-30.57	-2	-22	31
Sup. Frontal Gyrus	R	10	59	-7.34	26	57	9
Sup./Mid. Frontal Gyrus	R	10/9	402	-9.11	32	30	44
Mid. Frontal Gyrus	L	10	160	-12.96	-35	47	28
	L	11	61	-8.02	-26	45	-12
	L	9	162	-10.49	-23	40	41
	R	6	184	-10.07	14	-8	68
Insula	L	13	568	-12.54	-35	14	3
	R	13	783	-14.57	38	8	0
Mid./Inf. Temporal Gyrus	R	20/21	86	-11.38	59	-26	-20
Inf. Parietal Lobule	L	40	174	-9.83	-62	-31	30
	L	40	98	-10.68	-47	-62	42
English Positive							
Frontal, Temporal Gyrus	L		2641	20.29	-50	12	-21
Frontal, Temporal Gyrus	R		2293	15.82	53	25	20
Fusiform Gyrus	L	37/18	967	19.71	-20	-100	-17
	R	37/18	716	21.20	14	-94	-13
Sup. Frontal Gyrus	B	8	1133	15.40	2	42	51
Medial Frontal Gyrus	B	11	253	12.38	2	52	-12
Mid. Frontal Gyrus	L	6	34	5.42	-35	11	56
Precentral Gyrus	R	6	103	6.64	56	-9	32
Paracentral Lobule	R	5	74	7.30	4	-35	60
	B	7	224	16.29	-2	-62	26
	L	7	145	7.18	-29	-66	45
	R	7	99	6.25	26	-68	38
Inf. Temporal Gyrus	R	37	38	6.88	47	-64	-1
English Negative							
Cingulate, Precuneus, Lingual	B		5921	-31.19	-2	-22	31
Sup. Frontal Gyrus	L	9/10	518	-9.95	-32	31	40
	R	9	96	-7.72	41	21	40
	R	6	80	-6.24	11	-8	68
	R	6	46	-4.78	23	11	59
	R	10	384	-9.65	35	44	12
Insula	L	13	663	-12.16	-32	14	3
	R	13	835	-12.52	35	-8	-5
Precentral Gyrus	L	3	37	-5.16	-38	-19	44
Mid. Temporal Gyrus	R	21	54	-4.89	62	-29	-17
Inf. Parietal Lobule	L	40/41	286	-9.30	-56	-44	46

Notes: Sup. = Superior; Mid. = Middle; Inf. = Inferior; H = Hemisphere (L, Left; R, Right or B, Bilateral); BA = Broadmann's Area; Volume is given in number of voxels (2x2x2mm); Coordinates (x, y and z) for the peak active voxel in each cluster are given with reference to the MNI atlas.

Activity in many areas associated with reading (and language more generally) was strongly bilateral for both groups, as is often the case in comparisons of language tasks against rest. This pattern is likely to include activation related to a heterogenous collection of processes ranging from basic word recognition to sentence and discourse comprehension, along with attendant perceptual, memory, and reasoning processes. It is beyond the scope of the current analyses to disentangle these from one another, but we note that some discourse processes do seem to be right lateralized (see, e.g., [39]), whereas contrasts designed to identify reading-specific processes typically produce left-lateralized maps.

**Fig 2:**
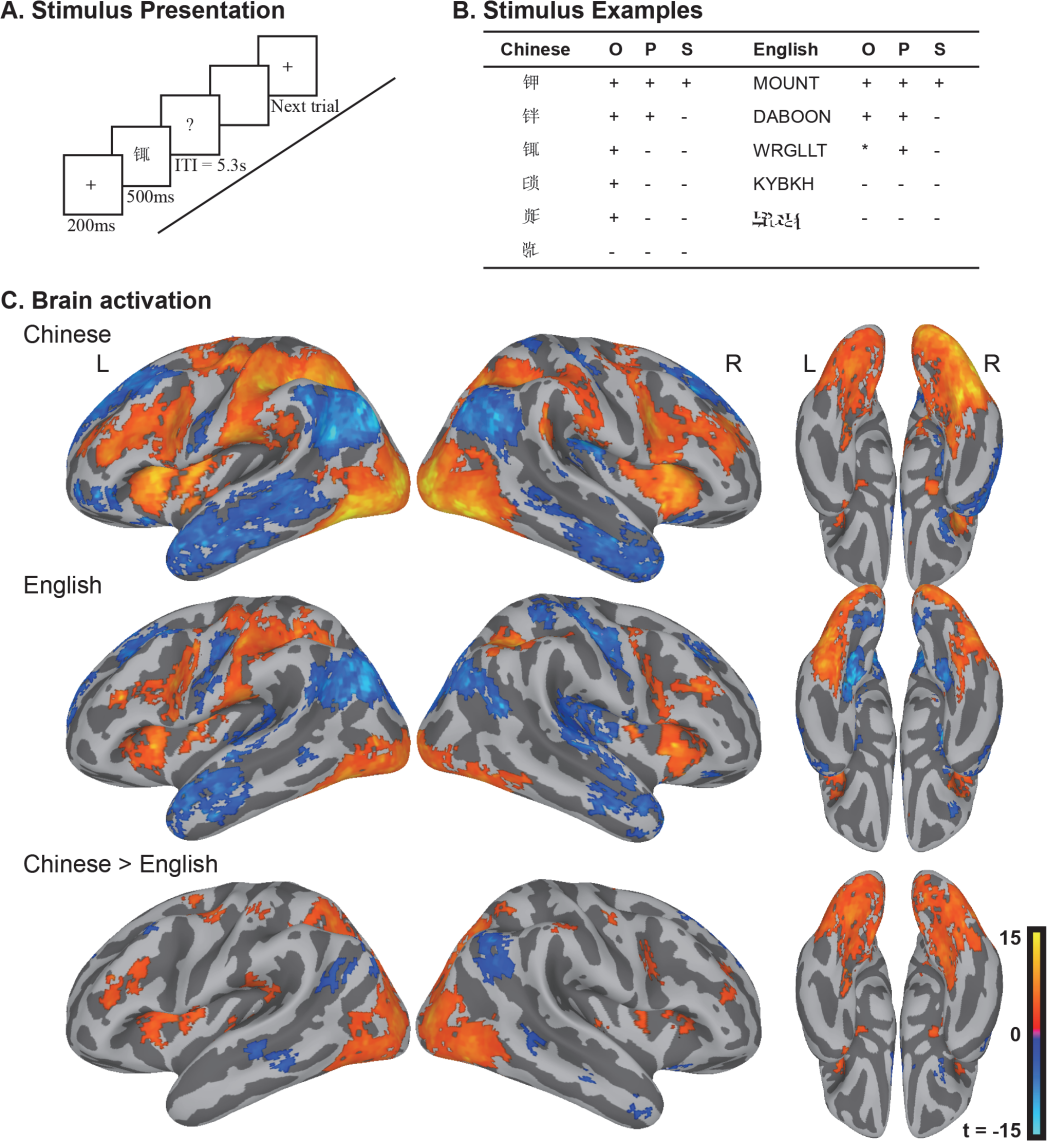
A. Event related designed were conducted for lexical decision task. Each trial began with a 200ms fixation. The stimulus was presented for 500ms with a blank screen for participants’ response. The mean ITI was 5.3s; B. Examples of each stimulus class designed for manipulating wordlikeness both for Chinese and English characters/words. C. The contrast brain map for Chinese > English.

Negative activations shown in Fig 2C and Table 2 are generally typical of "default mode network" (e.g., [40]) including precuneus, medial temporal cortex, posterior cingulate and anterior supramarginal gyrus. Deactivation was also observed throughout the insula. It is important to note that many functions associated with the default mode network—such as prospective memory and theory-of-mind reasoning (e.g., [41, 42])—are also potentially important for discourse comprehension. Thus, the large effect of greater activity during the rest periods than during reading, conceal more transient, positive activations throughout these regions associated with discourse comprehension.

#### Direct contrast between the two languages

A direct contrast between Chinese and English (Table 3) revealed robust differences in visual areas of the occipital and temporal lobes, consistent with the greater visual complexity of Chinese characters relative to English words. The precuneus is negatively activated in both languages, consistent with its participation in the default mode network, as discussed above; this region is more strongly deactivated for English than for Chinese, leading to a positive (Chinese > English) activation in the difference image. The region exhibiting this contrast is contiguous with inferior visual regions, where the positive activation is due to stronger activation in Chinese than in English.

**Table 3:** Regions identified as contrasting for Language in Experiment 1.

					Coordinates		
Region	H	BA	Volume	t	x	y	z
Chinese > English							
Lingual Gyrus	B	18	2060	9.05	4	-82	0
Temporal pole	L	38	68	5.50	-28	16	-30
	L	38	45	4.31	-38	4	-16
	L	38	33	4.44	-44	14	-10
	R	38	107	5.80	62	2	-4
	R	38	71	4.51	28	16	-30
Superior Temporal Gyurs	L	22	31	4.07	-62	-10	2
	R	22	49	6.00	64	-26	6
Middle Temporal Gyrus	L	21	66	5.74	-56	-52	-6
	L	20	23	5.38	-56	-10	-22
Inferior Temporal Gyrus	L	20	70	4.90	-34	-8	-28
	L	20	37	4.71	-40	-14	-24
	L	38	35	4.80	-44	4	-42
	R	20	27	3.84	34	-2	-40
Fusiform Gyrus	R	20	26	5.62	40	-22	-24
Precuneus	L	31	24	4.16	-16	-80	30
Middle Frontal Gyrus	L	6	29	5.15	-32	-4	54
vmPFC	R	25	51	6.65	2	38	-18
Insula	L	47	21	5.62	-26	10	-16
Cingulate	L	31	23	5.27	-2	-32	30
Parahippocampal Gyrus	L	20	22	4.31	-32	-20	-18
English >Chinese							
Anterior Cingulate	B		42	-6.21	2	1	16
Medial Frontal Gyrus	R	10	32	-4.57	14	44	15
Superior Frontal Gyrus	R	10	22	-5.72	26	59	22
Inferior Frontal Gyrus	R	0	27	-3.95	53	7	19
Superior Parietal Lobule	R	7	23	-5.00	32	-60	52

Note: H = Hemisphere (L,Left; R, Right or B, Bilateral); BA = Broadmann's Area; Volume is given in number of voxels (2x2x2mm); Coordinates (x, y and z) for the peak active voxel in each cluster are given with reference to the MNI atlas.

Activity in MTG, along with a number of semantic processing regions in temporal cortex, was also greater for Chinese than for English, consistent the view that reading in Chinese makes greater use of mapping from orthography to semantics than reading in English [7]. A small portion of precentral gyrus was also more strongly associated with Chinese than English, consistent with evidence for more automatic engagement of motor processes associated with writing during reading for complex characters than alphabetic text [43, 44]. Language differences were also observed in bilateral FFG: readers in both groups showed robust activity throughout the ventral occipital cortex, but this activity was stronger in Chinese than in English. In contrast with prior studies of language differences in reading, no differences were found in MFG or pST.

### Experiment 2: Lexical decision

The results of Experiment 1 are consistent with models in which the same cognitive processes are involved in reading for both English and Chinese. Such models predict that reading will engage a similar network of regions across languages, but with relatively greater dependence on semantic processing during Chinese reading, because of the statistical structure of mappings from orthography to phonology [7]. These data are difficult to reconcile with prior studies showing qualitative differences in the reading network between the two languages. We therefore hypothesized that language differences observed in prior studies might in fact reflect a language by task interaction; that is, differences in the apparent functional organization of the reading system between Chinese and English are largest during metalinguistic tasks. To test this hypothesis, in Experiment 2, we collected data from parallel lexical decision tasks conducted in English and Chinese. We first asked whether this task would replicate prior differences (showing main effects of language in MFG, FFG and pST), and then considered data from the two tasks in a factorial design to test directly for interactions between task and language.

#### Task vs. rest contrast for each language

In these lexical decision experiments, age-matched groups of native English and Chinese readers were presented with stimuli that varied in graduated levels of word-likeness from real words to scrambled text, and asked to decide on each trial whether the stimulus was a real word/character or not. As shown in Fig 2B, it is not entirely possible to match these stimulus manipulations across scripts. For this reason, and in order to parallel the analyses of the natural reading data, we tested for differences between the two languages in activity for task vs. rest.

Overall patterns of activity (Fig 2C, Table 4) included many of the reading-related regions identified in the naturalistic story reading, including bilateral vOT and inferior frontal gyrus (IFG) in both languages. Additional task-related activity is observed throughout precentral gyrus, likely due to the explicit motor demands of the lexical decision task. Consistent with our prior studies of metalinguistic tasks, we found robust activity in the insula to lexical decision task; this is of particular interest given that the insula was deactivated during naturalistic story reading, and may be related to the insula’s role in error-monitoring [45, 46].

**Table 4:** Regions identified in the Task > Rest contrast in Experiment 2.

					Coordinates		
Region	H	BA	Volume	t	x	y	z
Chinese Positive							
SFG,SPL,Occipital,Fusiform	B		21807	19.44	46	-64	-16
Middle/Inferior Frontal, Insula	R		3106	12.85	41	-6	13
Middle/Inferior Frontal, Insula	L		2868	12.38	-31	21	12
Inferior Parietal Lobule	R	40	166	8.78	64	-37	22
	L	40	144	6.07	-50	-42	28
Superior Temporal Gyrus	L	38	86	4.60	-25	7	-42
Thalamus			89	6.69	1	-12	10
Parahippocampal Gyrus	R	34	61	6.30	21	2	-17
	L	34	56	5.45	-23	4	-19
Chinese Negative							
Medial Frontal, Anterior Cingulate	B		6482	-18.02	1	20	-4
Posterior Cingulate Gyrus	B	31	1928	-14.78	-1	-60	25
Inferior Frontal Gyrus	L	45	302	-11.05	-37	52	5
Insula	R	13	258	-13.45	41	-22	18
Precentral Gyrus	L	4	98	-6.72	-62	-2	17
Postcentral Gyrus	R	3	484	-10.00	46	-29	62
Middle Temporal Gyrus	L	21	2278	-12.18	-39	25	-22
	R	21	2116	-9.98	60	-0	-17
Superior Parietal Lobule	R	7	74	-6.05	27	-49	60
Angular Gyrus	L	39	1304	-15.53	-41	-58	21
	R	39	734	-12.69	41	-75	29
Parahippocampal Gyrus	L		195	-11.14	-23	-16	-25
	L		77	-9.78	-27	-34	-12
	R		288	-10.43	31	-10	-27
English Positive							
Insula, pre-/post-central, IPL	L		2556	9.77	-54	-28	46
Fusiform Gyrus	R	37/18	2531	9.32	17	-89	-19
	L	37/18	2093	11.95	-15	-100	-15
Inferior Parietal Lobule	R	40	773	10.63	29	-67	42
Superior Parietal Lobule	L	7	50	6.16	-31	-56	62
Medial Frontal Gyrus	R	32	719	11.51	9	7	55
	L	32	624	9.89	-9	3	54
Middle Frontal Gyrus	R	9	142	7.77	46	24	30
	R	6	101	5.37	31	-6	54
	L	46	43	12.11	-39	29	28
Inferior Frontal Gyrus	L	6/9	670	9.42	-43	2	31
	R	9	210	6.39	41	2	33
	R	44	81	5.30	54	15	14
Precentral Gyrus	R	6	91	6.21	52	-3	41
Insula	L	13	530	10.85	-31	23	8
	R	13	480	12.44	33	23	8
Cingulate Gyrus	B	23	559	13.26	1	-2	30
English Negative							
Medial Frontal, Anterior Cingulate	B		2197	-11.94	-1	34	-3
Posterior Cingulate Gyrus	B	31	2832	-18.93	-1	-60	21
Superior Frontal Gyrus	L	8	607	-11.12	-25	24	40
Medial Frontal Gyrus	R	6	203	-7.31	13	-18	45
Middle Frontal Gyrus	R	8	201	-8.62	23	26	45
	L	6	119	-9.06	-37	9	55
Inferior Frontal Gyrus	R	47	63	-7.13	37	37	-12
Precentral Gyrus	L	4	189	-7.64	-37	-20	40
	R	4	62	-4.86	11	-35	72
Postcentral Gyrus	R	3/4	1284	-12.73	35	-28	44
Superior Temporal Gyrus	R	22	2282	-10.66	46	-10	4
	R	38	61	-5.67	21	24	-44
Middle Temporal Gyrus	L	21	1242	-19.09	-41	-16	1
Precuneus, Angular Gyrus	L	19/39	1510	-11.93	-43	-74	24
Angular Gyrus	R	39	436	-11.55	50	-55	14
Precuneus	R	7	88	-7.83	9	-70	55
Cuneus	R	18/19	466	-10.24	23	-86	41
Lingual Gyrus	L	18	97	-8.67	-7	-77	-14
	R	18	72	-7.84	11	-75	-9
Parahippocampal Gyrus	L		719	-18.40	-31	-36	-14
	R		305	-12.67	25	-4	-22
	R		246	-10.61	27	-36	-12

Note: H = Hemisphere (L,Left; R, Right or B, Bilateral); BA = Broadmann's Area; Volume is given in number of voxels (2x2x2mm); Coordinates (x, y and z) for the peak active voxel in each cluster are given with reference to the MNI atlas.

Deactivation was observed in the angular gyrus, and in the left superior temporal sulcus (this deactivation was restricted to the anterior portion of STS for English, and was more extensive in Chinese). This again contrasts with the results of the naturalistic reading experiment, in which STS was strongly activated for both languages. The STS, especially its anterior aspect, is typically associated with semantic processing [47] but we have observed it to be negatively correlated with the reading network in other laboratory tasks that do not explicitly require semantic processing [22]. The dynamics of activation in this area during lexical decision are an interesting question for future research, given the influence of semantic variables on behavior in this task [17].

#### Direct contrast between the two languages

A direct contrast between the two languages (Fig 2C, Table 5) reveals limited similarities between the lexical decision and naturalistic reading data in Experiment 1: large differences between Chinese and English are observed in visual areas of the occipital and temporal lobes. Otherwise, the results more closely resemble those of prior comparisons between the two languages based on data from meta-linguistic decision tasks. Unlike naturalistic reading, lexical decision was associated with language differences in the mid-fusiform (putative VWFA) bilaterally, and the MFG (also bilaterally). In both of these cases, differences reflected greater and more extensive activation for Chinese than for English.

**Table 5:** Regions identified as contrasting for Language in Experiment 2.

					Coordinates		
Region	H	BA	Volume	t	x	y	z
Chinese > English							
Fusiform Gyrus	B	37/18/19	9280	10.55	-1	-65	3
Cingulate	B	24	630	7.90	1	8	35
Inferior Frontal Gyrus	R	9	112	4.36	41	4	28
Precentral Gyrus	L	6	203	6.78	-43	-12	56
	L	6	109	5.34	-48	-6	8
	R	4	47	5.10	43	-18	56
Postcentral Gyrus	L	40	48	3.94	-43	-36	50
Insula	L	44	406	6.26	-33	11	12
	L	13	104	5.61	-48	-27	20
	L	41	72	5.29	-39	-33	16
	R	13	81	4.30	33	27	12
	R	13	69	4.71	35	7	11
	R	13	64	5.14	39	-20	-1
	R	13	58	5.81	41	-6	13
Medial Frontal Gyrus	L	6	105	5.21	-7	-16	58
	R	6	63	4.98	15	-15	69
	L	9	82	4.52	-41	20	34
	L	46	80	5.95	-41	33	30
	L	6	68	5.69	-19	-6	63
	R	9	62	5.39	27	41	33
	R	10	52	4.42	39	41	24
Superior Temporal Gyrus	L	38	43	6.18	-27	18	-47
	R	38	43	4.52	25	11	-40
Superior Parietal Lobule	R	7	43	4.89	7	-70	51
	R	40	73	4.73	56	-36	46
Precuneus	L	7	67	5.72	-11	-66	55
	L	7	55	4.26	-9	-78	52
	R	7	49	5.65	29	-59	49
Parahippocampal Gyrus	L	35	71	7.45	-33	-33	-16
	L	34	49	5.05	-21	-2	-22
	R	34	44	5.11	19	-0	-17
Claustrum	L	13	48	4.86	-39	-20	-4
English > Chinese							
Anterior Cingulate	B		462	-9.30	1	24	-15
Cingulate Gyrus	B		86	-7.04	-1	-22	40
Superior Frontal Gyrus	R	8	181	-5.79	11	46	46
	R	8	69	-4.77	15	27	56
	L	8	48	-4.98	-11	48	44
Medial Frontal Gyrus	R	9	62	-6.52	9	45	33
	L	10	45	-5.17	-9	46	5
Middle Frontal Gyrus	L	8	42	-4.62	-37	17	49
Inferior Frontal Gyrus	R	47	73	-5.04	27	24	-18
Middle Temporal Gyrus	L	21	84	-5.23	-58	-30	-9
	L	21	51	-5.18	-50	-23	-8
	R	21	80	-4.73	62	-38	-5
	R	21	78	-4.88	54	11	-35
	R	21	43	-4.57	64	-11	-12
Angular Gyrus	L	39	89	-4.81	-46	-66	23
	R	39	276	-7.84	50	-63	36
Precuneus	L	19	54	-4.89	-39	-75	42
	L	19	51	-5.71	-37	-81	28

Note: H = Hemisphere (L, Left; R, Right or B, Bilateral); BA = Broadmann's Area; Volume is given in number of voxels (2x2x2mm); Coordinates (x, y and z) for the peak active voxel in each cluster are given with reference to the MNI atlas.

The language difference observed in MTG is due to a greater deactivation relative to rest observed for Chinese readers. Note that this location in MTG is anterior to the region associated with orthographic to semantic mapping that was found in the naturalistic task. A similar pattern of deactivations explains the contrast in AG. As in Experiment 1, the large contiguous "blob" encompassing inferior/lateral portions of the occipital lobe, along with the precuneus and other superior midline areas in the difference image comprises two different kinds of effect. The midline regions are strongly deactivated in English, but are neither activated nor deactivated in Chinese. The more lateral visual regions typically associated with reading-related processes show greater activation for Chinese than English (without a difference in sign) and more bilateral activity in Chinese than English, consistent with prior fMRI studies of laboratory reading tasks. In addition, stronger activity is observed in superior parietal, and the post- and precentral gyri for Chinese compared to English. Thus, in striking contrast to the data from Experiment 1, a task requiring a meta-linguistic decision gave rise to robust differences between groups in regions that have previously been identified as specific to Chinese.

#### Language-by-task interaction in whole brain analyses

**Fig 3:**
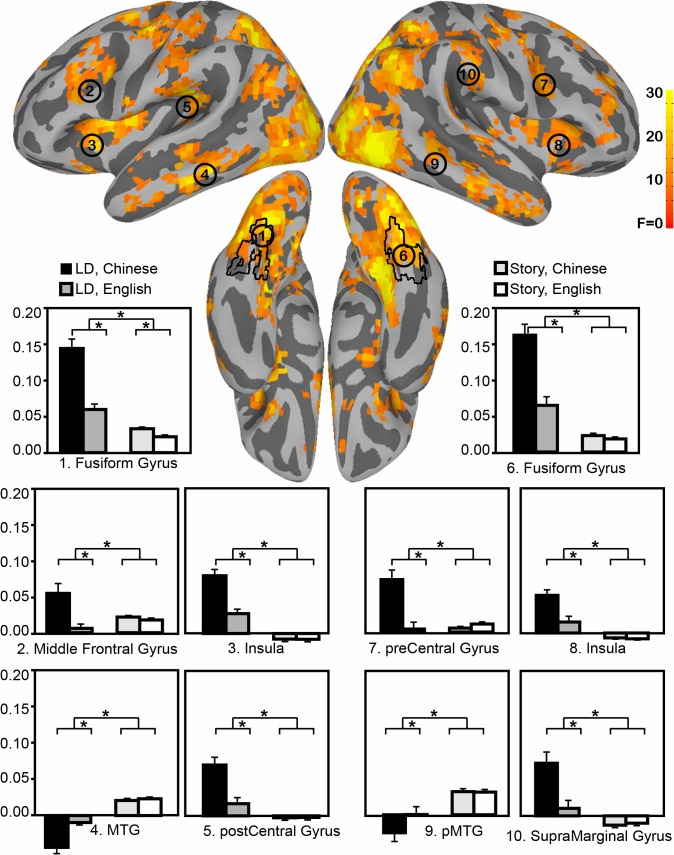
Brain regions identified as interaction of Task by Language. The black outlier indicates anatomical mask of bilateral fusiform gyrus. The bar graphes plot each ROI’s activity pattern.

We conducted a whole brain ANOVA to test for Language x Task interactions (Table 6, Fig 3). Significant interactions were observed in a large cluster of bilateral regions including visual cortex, precuneus, superior parietal lobule, angular gyrus and fusiform gyrus, as well as a number of regions at superior pre-/post-central gyrus, supramarginal gyrus, anterior and middle temporal gyrus, middle frontal gyrus and insula.

**Table 6:** Regions identified as interaction of language-by-task.

			Corrdinate		
Region	Volumes	F	x	y	z
Bilateral Hemisphere					
Visual Cortex, FFG, Precuneus, SPL	4163	62.66	29	-85	-2
Cingulate Gyrus	1368	40.46	-44	-13	54
Left Hemisphere					
Precentral Gyrus	352	41.75	-35	10	10
	142	20.53	-35	3	36
Superior Frontal Gyrus	110	20.27	-4	52	38
Inferior Frontal Gyrus	98	24.67	-32	21	-21
Insula	140	33.66	-47	-27	21
Postcentral Gyrus	72	22.67	-50	-34	37
Middle Temporal Gyrus	143	26.71	-62	-22	-20
	62	16.66	-53	6	-32
Cingulate Gyrus	112	37.40	-2	-22	38
Parahippocampal Gyrus	59	23.16	-26	-1	-15
Right Hemisphere					
Superior Frontal Gyrus	167	25.10	14	49	48
Medial Frontal Gyrus	77	26.44	8	-17	64
Insula	346	32.30	41	-5	16
Precentral Gyrus	212	22.99	41	3	29
Middle Frontal Gyrus	64	21.70	41	41	24
	56	30.58	29	40	34
Postcentral Gyrus	85	19.73	56	-35	47
	60	15.50	62	-28	24
Middle Temporal Gyrus	311	28.06	53	9	-39
	70	19.61	62	-36	-3
Declive	51	40.75	17	-75	-19
Parahippocampal Gyrus	50	19.53	20	-1	-11

Note: Volume is given in number of voxels (2x2x2mm); Coordinates (x, y and z) for the peak active voxel in each cluster are given with reference to the MNI atlas.

Two types of Language x Task interaction were observed (Fig 3). One group of regions activated stronger for Chinese than English during lexical decision task, while they showed a smaller or null language effect during naturalistic reading. Those regions included FFG, MFG, insula, pre/post-central gyrus, as well as a region near anterior supramarginal gyrus. Another group of regions, including posterior MTG and angular gyrus, are deactivated for Chinese LDT, but less deactivated for English LDT, whereas during naturalistic reading they are positively activated in both languages, with no difference between Chinese and English.

#### Comparison of naturalistic and artificial task data

**Fig 4:**
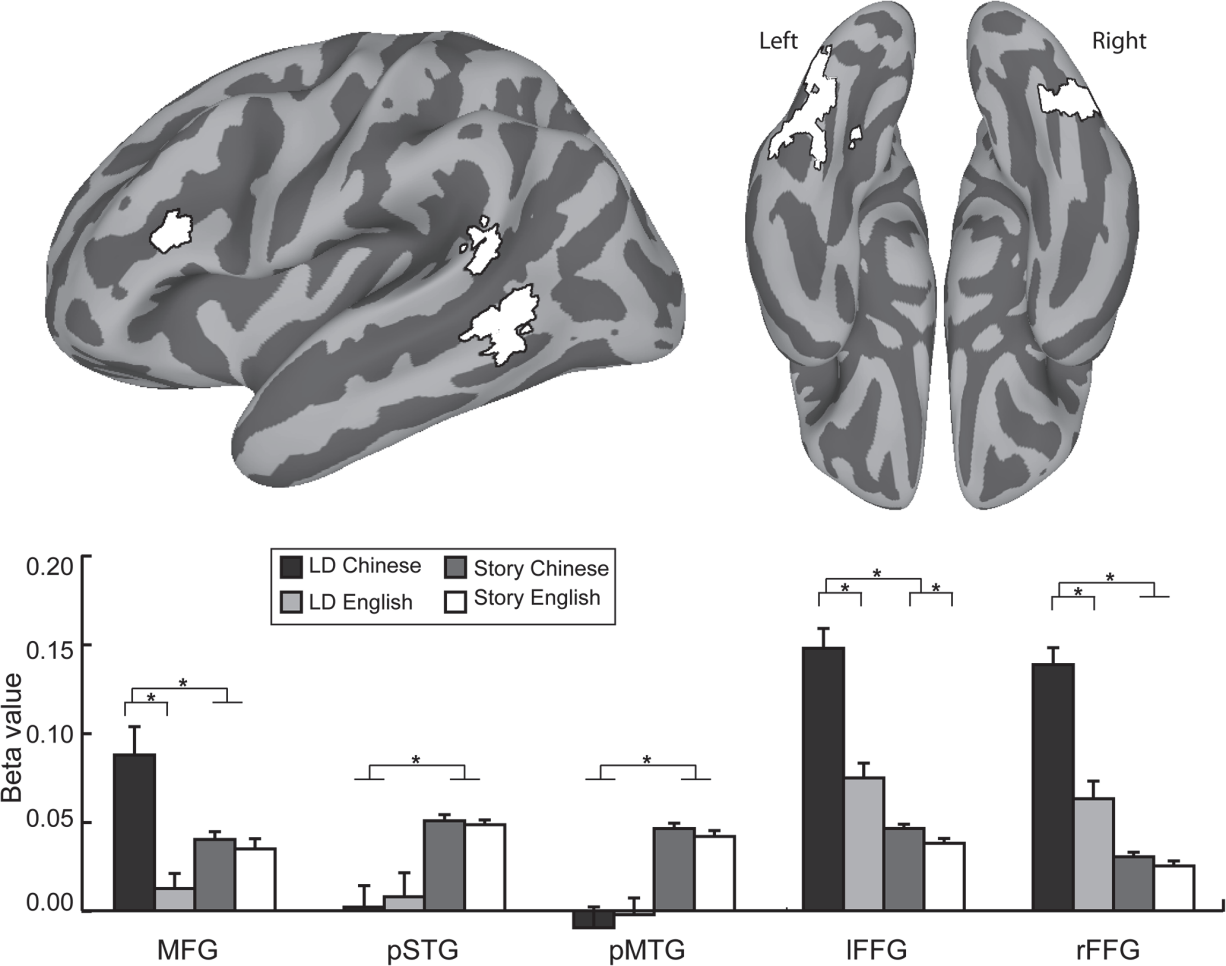
Data from lexical decision (LD) and story reading in ROIs from meta-analyses of Chinese and English reading. Large language by task interactions are observed in middle frontal gyrus (MFG), and left and right fusiform gyrus (FFG), indicating that language differences are exaggerated by artificial task conditions in these regions. In posterior superior and middle temporal gyri (STG and MTG), only a large main effect of task was found, suggesting that these regions are engaged more strongly by natural reading conditions than lexical decision in both languages.

The whole-brain analyses revealed a very large network of regions that produce a Language x Task interaction. In order to test whether specific regions identified in the literature show this interaction, we selected a set of regions of interest from meta-analyses of the Chinese reading literature, and tested for the same interaction based on ROI analyses (Fig 4). Regions were selected based on prior meta-analyses that have identified differences between alphabetic and morphosyllabic scripts [11, 21], and a meta-analysis of Chinese reading [33].

Interactions in which language effects are seen only during the lexical decision task imply that language differences in the tested region are related to meta-linguistic task demands. This pattern was found in the MFG, where the Language x Task interaction was reliable *F* (1,56) = 11.29, *p* <. 005. The effect of Language for lexical decision was significant, *F* (1,28) = 18.12, *p* <. 001, but the Language effect for naturalistic reading was not, *F* (1,28) < 1. A similar pattern was observed in right FFG, Language x Task, *F* (1,56) = 25.78, p <. 001, Language effect for lexical decision, *F* (1,28) = 40.36, *p* <. 001, Language effect for naturalistic reading, *F* (1,28) = 2.11, p =. 15.

In the left FFG, there was a significant Language by Task interaction, *F* (1,56) = 151.35, *p* <. 001. Although significant Language effects were found in both contexts, with Chinese greater than English, the Language effect for lexical decision, *F* (1,28) = 46.84, *p* <. 001 was nonetheless stronger than for naturalistic reading, *F* (1,28) = 5.60, *p* =. 025. Thus, although the effects are much larger under task demands that bias orthographic processing, small but reliable differences are observed in this region during naturalistic reading, consistent with the both the greater visual complexity of the stimuli [48] and their more complex mappings to phonology and semantics [18].

The ROI identified in left pST, when mapped to the surface, produced distinct regions in STG and MTG. Because these regions are thought to be functionally distinct, an anatomical mask was used to divide this ROI into superior and middle temporal portions. In both of these ROIs, a very different pattern was observed from the MFG and FFG, such that there was no interaction between Language and Task, Fs < 1. Instead, a main effect of Task was observed in both STG, F (1, 56) = 21.73, p <. 001, and MTG, F (1,56) = 40.59, p <. 001, such that activity was greatest during naturalistic reading, and did not differ between the two groups. This region is associated with mapping from spelling to sound in alphabetic languages [9] and is rarely observed to be active in studies of Chinese character recognition (but see [23]). The lack of activity in this region during lexical decision for both languages is not entirely surprising, given the relatively weak demands that task puts on mapping from orthography to phonology [49].

## Discussion

The overall pattern of results from naturalistic reading and lexical decision reveals substantial flexibility in the deployment of cortical resources in response to task demands. Dramatic differences in brain activity were observed across task. For example, much of the superior temporal sulcus, supramarginal and angular gyri were strongly activated during story reading, but deactivated during lexical decision. These regions are associated with various aspects of language processing, and seem to interact in important ways during spoken and written language comprehension [50–52] and thus it is not surprising to see their activity associated with a task that involves comprehension of an extended discourse.

In contrast, within-task differences between languages were relatively subtle, generally reflecting a differential degree of activity in the same network across writing systems. Further, very different patterns of language-specificity were observed across the two tasks. Language by task interactions were observed in regions previously identified as playing a specific role in Chinese reading [11, 12, 53, 54]. Comparisons of language differences across tasks are of particular interest, because thus far our understanding of the brain network for reading—and thus how it may differ across writing systems—has been based almost entirely on data from artificial tasks. Language differences in the reading network need to be understood in the context of interactions between task and stimulus properties [18–20], and we have shown here that naturalistic reading studies can add valuable data to this discussion.

During naturalistic reading, differences between English and Chinese were observed throughout the visual cortex, in the left middle temporal gyrus and ventral occipitotemporal regions bilaterally. Differences in visual regions are likely related to the greater visual complexity of Chinese characters relative to English words [12, 21, 54, 55].

Language differences in MTG are in line with the notion that mapping from orthography to semantics plays a more important role in Chinese character recognition than in English word recognition [13, 56]. This difference in affordances for orthography-to-semantics mappings arises because Chinese characters contain probabilistic cues to meaning that are unrelated to pronunciation [1]. Activity in the MTG has been shown to be task-dependent. For example, Booth et al. [13] observed MTG activity during a semantic judgment task, but not during a closely matched phonological judgment task (see also [57, 58]). Thus it is interesting to note that this region is strongly activated in both languages during natural reading, and that language differences are predictable from differences in the relative demands of each script on direct mapping from orthography to semantics [7, 23].

As in the story reading task, language effects in the lexical decision task were observed in visual regions. Aside from this, the differences between the brain networks for reading in English and Chinese differ sharply from what is observed under naturalistic reading. Activity in the portions of the ventral temporal cortex (fusiform gyrus or FFG) associated with visual processing were generally more strongly activated during both tasks for Chinese than for English. An anterior portion of particularly is strongly associated with visual word recognition [18, 59]. Region of interest analyses of the left and right FFG revealed strong language by task interactions. Activity in this region was greater overall for lexical decision in both hemispheres, and this difference was exaggerated for Chinese readers relative to English readers.

Robust activity was observed in an anterior portion of the middle frontal gyrus for Chinese, whereas activity in this region was weaker and less extensive for English. This is consistent with prior research using artificial tasks such as phonological and semantic similarity judgment [12, 13, 34, 60]. Activity in this region is similar across languages during the story reading task. Investigation of a region of interest based on meta-analyses of Chinese and English reading revealed a large language by task interaction in the middle frontal gyrus, consistent with the notion that language differences in this region may be driven by the meta-linguistic decision-making demands of this task, or by visual processing that is particularly important for processing Chinese characters in isolation [11, 34].

The notion that MFG is related to visual processing demands is supported by evidence that it is strongly activated during lexical decision tasks in Korean, a transparent alphabet that has in common with Chinese only its visually dense and non-linear character structure [54, 61]. On the other hand, we show here that MFG activity is strongly modulated by task (see also BOOTH, etc.). Analysis of effects of stimulus class [19] shows that activity in the MFG tracks with behavioral measures of difficulty during LDT. Notably, stimulus effects are not observed in MFG for the same stimuli in a one-back task [22], which arguably puts an even greater premium on visual processing, but requires less in the way of meta-linguistic decision-making. Thus, we would suggest that the data so far are most consistent with view that the specific activation of MFG for Chinese is related to an interaction between the visual complexity of the orthography, and task demands related to meta-linguistic decisions. These results may have implications for the relationship between MFG activity and dyslexia in Chinese, which, given the task-dependency of MFG activation, might be explained in terms of struggling readers’ relative inability to flexibly deploy different components of the reading network in the face of task demands [13], rather than to a constitutive deficit localized to MFG. In any event, comparisons of results from naturalistic reading tasks in struggling readers across languages are likely to reveal interesting language differences.

Activity in the posterior superior temporal cortex is often associated with mapping from orthography to phonology in alphabetic languages [10, 37, 62, 63]. The lack of activity in this region during reading tasks in Chinese is often attributed to the lack of “assembled phonology” [3, 11]. That is, mappings from written to spoken forms in Chinese are not componential in the way that alphabetic representations are. Instead, whole characters, or their phonetic components, are probabilistically associated with whole syllables [3]. This region was not activated during lexical decision in either language. Further, analyses of these data by Yang & Zevin [64] revealed no activity in pST in analyses of task vs. rest, nor did we find evidence for sensitivity to specific stimulus classes. This area was equally active in both languages during naturalistic reading, however. This is difficult to interpret, however, because the pST is involved in a wide range of language processes that are engaged by understanding coherent narratives [65] in addition to its role in spelling-to-sound mappings.

The whole brain GLM analyses show a very large Language x Task interaction in the insula as well, such that the language difference is restricted to the LD task. That activity is restricted to the LD task is not surprising, given the insula’s role in error monitoring [45, 46], and the language difference may be explained in terms of the lower overall accuracy in the Chinese behavioral data. Yang et al. [19] and Yang & Zevin [64] further showed that insula activity to conditions with the lowest accuracy in the lexical decision task in both languages. This is a perfect example of a region that behaves very differently under artificial task conditions and naturalistic reading.

Thus, throughout the reading network, and particularly those regions thought to differ in skilled Chinese and English readers, there are important differences between the patterns that emerge under different task demands. This does not seem to be due to differences in the overall power to resolve contrasts across tasks; consideration of the peak inferential statistical values and overall extent of activation in Tables 2 and 4 suggests that the block design used in Experiment 1 produces much stronger contrasts than Experiment 2, and yet between-language contrasts in left MFG and bilateral FFG are greatest in Experiment 2. Instead, it seems as though the overall reorganization of patterns of brain activity in response to task demands can interact with the affordances specific writing systems provide for meeting those demands.

In Chinese, it is possible to construct pseudo-characters that have cues to both phonology and semantics, whereas in English, pseudo-words based on the monomorphemic words used in most studies contain no cues to meaning. In English, it is not possible to create orthographically word-like strings that do not have a plausible pronunciation, whereas in Chinese this is easily done. A sizeable minority of Chinese characters contains neither cues to meaning nor cues to pronunciation, and so it is possible to construct pseudo-characters that also lack these cues, but are orthographically legal. Thus, the stimulus parameters that are available for manipulation by experimenters differ greatly between the two languages. Whereas English readers can distinguish words from non-words by pooling information about spelling, pronunciation and meaning at multiple levels of description, Chinese readers are much more dependent on determining whether they recognize the particular orthographic configuration at the whole character level.

This notion, that the different writing systems have different task-specific affordances, contrasts with the view that differences between patterns of brain activity observed in reading tasks reflect cultural differences in brain organization resulting from experience with different scripts. Reading connected text silently for meaning more directly simulates the behavior we wish to understand by studying reading in the brain than making metalinguistic decisions about isolated words or characters. But it is not necessarily the case that the language differences we observed in our “naturalistic” task are the “real” differences between the reading networks in the two languages. During both silent reading and lexical decision, a number of ancillary cognitive and linguistic abilities are active simultaneously, creating ambiguity about structure-function relationships across languages. For example, differences between Chinese and American English readers in midline regions and the inferior temporal lobe, well outside the typical reading network, may plausibly be related to cultural differences in narrative comprehension, as these regions are associated with discourse comprehension [66]. The activity observed in the posterior STS is also difficult to interpret. Is there some probabilistic orthography-to-phonology mapping that is, for some reason, more likely to be engaged during processing of connected text? Or is the activity observed during story reading more related to general language comprehension?

What is clear from these data is that brain activity is organized very differently during different tasks, and that language effects can interact with task. A full understanding of the reading network—and how, or whether it differs in important ways between languages—will require interpretation of main effects of language in the context of potential interactions with task demands.
